# Use of Fly Screens to Reduce *Campylobacter* spp. Introduction in Broiler Houses

**DOI:** 10.3201/eid1312.070488

**Published:** 2007-12

**Authors:** Birthe Hald, Helle M. Sommer, Henrik Skovgård

**Affiliations:** * Technical University of Denmark, Aarhus, Denmark; † Technical University of Denmark, Søborg, Denmark; ‡ University of Aarhus, Lyngby, Denmark

**Keywords:** *Campylobacter*, flies, chickens, poultry, intervention study, seasonal variation, risk reduction, human, campylobacteriosis, food contamination, dispatch

## Abstract

Fly screens that prevented influx of flies in 20 broiler houses during the summer of 2006 in Denmark caused a decrease in *Campylobacter* spp.–positive flocks from 51.4% in control houses to 15.4% in case houses. A proportional reduction in the incidence of chicken-borne campylobacteriosis can be expected by comprehensive intervention against flies in broiler production houses.

Campylobacteriosis is a severe gastroenteric human disease of global significance. The incidence correlates with the prevalence of thermophilic *Campylobacter* spp., predominantly *C. jejuni* and *C. coli* (*1*), in chickens and follows a seasonal cycle in temperate climates for reasons not fully elucidated. The number of cases is lowest in winter and highest in summer (*2*). In Denmark, the prevalence of *Campylobacter* spp.–infected chicken flocks peaked at 60%–80% in recent summers (*3*). The population size of flies displays a similar cycle (*4*). Flies, in particular the house fly, *Musca domestica*, are well-known vectors of several enteric bacterial diseases (*5*) and are known to carry *Campylobacter* spp. (*6**–**10*). Vector flies can transmit *Campylobacter* spp. from outside farm livestock to broiler flocks because large numbers of flies may enter broiler houses by ventilation air (*7**,**11*). Our aim was to evaluate the effect of insect screens in addition to existing biosecurity measures against *Campylobacter* spp. infection of broiler chickens in summer.

## The Study

**Figure 1:**
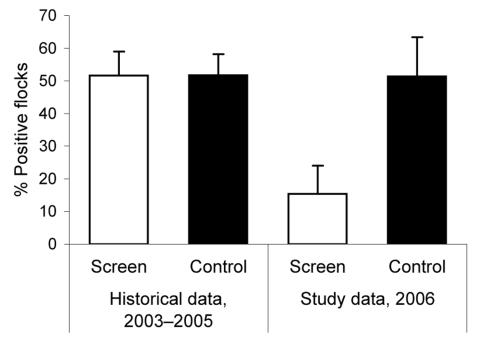
Percentage of *Campylobacter* spp.–positive broiler flocks produced in fly screen houses and control houses June 1 to November 13 during 2003–2005 (historical data) and in 2006 (during intervention).

Potential study sites were identified in the Danish Poultry Council´s national surveillance database (*3*) on the basis of the number of *Campylobacter* spp.–positive flocks produced in broiler houses during 2003–2005. All farms practiced hygiene procedures such as separating clean and dirty zones, changing footwear and clothes, and washing hands with disinfecting soap before entering the broiler room. Furthermore, a 3-m zone with short-cut grass or gravel surrounded the houses. Houses were emptied, cleaned, and dried before each new flock of chickens was brought in. All farmers were instructed to maintain biosecurity and management routines as before the study. Case and control groups were assigned to match each other in *Campylobacter* spp. prevalence and were composed so that the distribution of previous *Campylobacter* spp. prevalence of flocks for each group (June to November during 2003–2005) were equal (Figure 1) and with similar distribution in the presence of other livestock in a periphery of 1.5 km around the farms. Farmers consented to participate before study groups were composed.

According to data from the national Danish *Campylobacter* surveillance program (*3*), the historical *Campylobacter* spp. prevalence at slaughter during 2003–2005 from June to November had been 51.6% (95/184) (95% confidence interval [CI] 44.3%–59.0%) in case houses and 51.7% (123/238) (95% CI 45.2%–58.2%) in control houses. Thus, before the study, the baseline prevalence for houses in the case and control groups were not significantly different from each other (p = 0.99 by χ^2^ test).

Twenty houses on 11 farms in Jutland, Denmark, were equipped with fly screens by June 1, 2006 (photographs available from www.vet.dtu.dk/default.aspx?id=20832). Fifty-two broiler flocks stocked in the houses after June 1 constituted the cases; the last flock was slaughtered on November 6, 2006. Controls were 70 broiler flocks reared in 25 matched broiler houses on 13 other farms without fly screens; the last flock was slaughtered on November 13, 2006. All houses were ventilated through wall inlets in the long sides of the houses, air outlets through chimneys in the roofs, and gable fans. The study design was based on experience gained in a pilot study in 2004 (*11*) of 5 farms with parallel case and control houses on each farm. The pilot study showed a significant delay of *Campylobacter* spp. introduction in case houses. However, only a 37% reduction in positive broiler flocks was obtained at slaughter due to transmission of *Campylobacter* spp. from control houses to the corresponding case houses.

Broiler flocks were sampled at days 21, 28, and 35. Boots with over-shoe covers were used to walk through the broiler rooms. The over-shoe covers (photographs available from www.vet.dtu.dk/default.aspx?id=21756) were analyzed for *Campylobacter* spp. Results are shown in Table 1. Flocks were slaughtered between days 35 and 42 and sampled by collection of 10 cloacal swabs per flock at the abattoir. Results of the current national surveillance program of *Campylobacter* spp. in broiler production were included in the study as reference to ordinary Danish broiler production. All samples were analyzed by PCR (DANAK [The Danish Accreditation and Metrology Fund] accredited method) detecting thermophilic *Campylobacter* spp. (*12*).

**Table 1:** *Campylobacter* spp. positive and negative flocks by type of house

Type of house	Day 21			Day 28			Day 35	
	No. positive (%)	No. negative		No. positive (%)	No. negative		No. positive (%)	No. negative
Fly screened (n = 52)	3 (5.8)	49		3 (5.8)	49		4 (7.7)	48
Control (n = 70)	8 (11.4)	62		20 (28.6)	50		30 (45.5)	36

**Figure 2:**
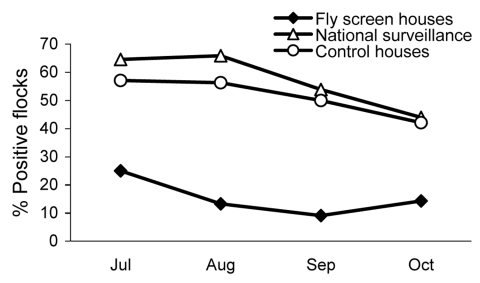
Prevalence per month of *Campylobacter* spp.–positive broiler flocks during the study period (June 1–November 13, 2006) in fly screen houses (52 flocks) and in control houses (70 flocks), and the national flock *Campylobacter* spp. prevalence at slaughter of 1,504 flocks according to surveillance data for the same period.

In fly screen houses (case houses), 15.4% (95% CI 7.7%–27.8%) of the flocks reared during the study period were *Campylobacter* spp. positive at slaughter, whereas the prevalence in *Campylobacter* spp.–positive flocks reared in the control houses was 51.4% (95% CI 40.0%–62.7%). The prevalence in the control houses remained unchanged (p = 0.68 by χ^2^ test) compared with the historical prevalence during June–November, 2003–2005. Figure 1 shows the *Campylobacter* spp. prevalence of flocks from the study with the historical data. The average flock *Campylobacter* spp. prevalence per month in 2006 in fly screen houses and in control houses is shown in Figure 2 with the results of the national Danish *Campylobacter* surveillance program of 1,504 broiler flocks slaughtered in Denmark in specific months.

Data were analyzed with SAS software (SAS Institute, Cary, NC, USA) in the SAS procedure proc genmod with a logit link function and a repeated statement where subject = flock. The repeated statement accounts for the intraclass correlation. In the model, the effects of the fly screen “Screen” of the time between 21 and 35 days “Time”, the interaction “Screen Time” and the effect of the average monthly prevalence level at slaughter “Month” (analyzed as regressor) were analyzed. The status at day 35 was chosen in the analysis instead of the results at slaughter to avoid biases in data due to the increased risk of introducing *Campylobacter* spp. in those flocks slaughtered later and during depopulation and transportation to slaughter. Only 4 flocks were slaughtered during November and were merged with the October flocks in the analysis.

The analysis shows a clear effect of fly screens (p = 0.0002) by either complete prevention of infection or by a significant (p<0.0001) delay in onset of infection of the broiler flocks. Results of analyzed sources and estimates of flock *Campylobacter* spp. status in fly screened and in unprotected houses at days 21 and 35 predicted by the applied statistical model are shown in Table 2.

**Table 2:** Results of analyzed sources and estimates of flock *Campylobacter* spp. status from the applied statistical model

Type of result	p value
Source of variation*	
Screen	0.0002
Time (day of rotation)	<0.0001
Screen time	0.07
Month	0.80
Predicted prevalence of *Campy­lo­bacter* spp.–positive flocks (day 21/35), %	
Fly screen houses	3/11
Houses without fly screens	14/42

*Analysis of variance, type 3 test. Significant effects if p<0.05.

## Conclusions

We showed that preventing flies from entering broiler houses in the summer of 2006 caused a drop in prevalence of *Campylobacter* spp.–positive flocks at slaughter from 51.4% in control houses to 15.4% in case houses. It seems reasonable that the main results found in this study can be extrapolated to the national situation because the selected control houses had a prevalence similar to the national prevalence level for the same period (Figure 2). Installation of effective fly screens in broiler houses in Denmark would most likely decrease the average yearly *Campylobacter* spp. prevalence, and show a major decrease in the summer peak. Presumably, the risk for infection from eating chicken, the main cause of campylobacteriosis in Denmark (*13*), would be reduced. The expected effect on the incidence of chicken-borne campylobacteriosis has been calculated by Rosenquist et al. (*14*) to be proportional to the decline in flock *Campylobacter* spp. prevalence.

Our study provides evidence that flies are vectors for *Campylobacter* spp. in broilers and furthermore, probably explains the seasonal variation of *Campylobacter* spp. in chicken products. Flies may also play a role in direct transmission of *Campylobacter* spp. to humans (*14**,**15*). Certainly, the issue deserves further scientific investigation.
